# Inferior colliculus microcircuits

**DOI:** 10.3389/fncir.2014.00113

**Published:** 2014-10-09

**Authors:** Manuel S. Malmierca, Eric D. Young

**Affiliations:** ^1^Auditory Neurophysiology Unit, Laboratory for the Neurobiology of Hearing, Institute of Neuroscience of Castilla y León, University of SalamancaSalamanca, Spain; ^2^Department of Cell Biology and Pathology, Faculty of Medicine, University of SalamancaSalamanca, Spain; ^3^Neural Encoding Laboratory, Biomedical Engineering, Johns Hopkins UniversityBaltimore, MD, USA

**Keywords:** inferior colliculus, neural pathways, internal circuitry, inhibitory circuits, ion channel models, representation of sound, sonar

A unique aspect of the auditory system is the inferior colliculus (IC). This large midbrain structure serves as an obligatory synaptic station in both the ascending and descending auditory pathways. It has no equivalent in other sensory systems and it meets several unique needs of the auditory system. Most important, of course, is unifying the representation of sound in the two ears, which allows sound localization and other spatial calculations such as demasking and the analysis of complex auditory scenes. In addition, the IC is a major target for non-auditory inputs to the auditory system, including connections from other sensory systems and from neuromodulatory systems like the locus coeruleus. The IC also distributes auditory information in cortico-cortical loops and in connections to the superior colliculus.

There is much to be learned about the IC. The goal of this special topic is to bring together papers, both reviews and original research, on a wide range of aspects of the IC's organization and function. These include the internal and external connections of the IC, the molecular determinants of its response properties, and the nature of sound encoding in the IC. In this e-book, 31 contributions are organized into these three broad categories.

The first 12 chapters address the morphological and functional organization of the IC. They include descriptions of the connections of the IC to other parts of the brain, both ascending and descending projections, as well as the organization of internal microcircuits within the IC itself. In most brainstem auditory centers, the neurons have a diverse array of morphological and molecular structure which correlates strongly with connectivity and functional role (inhibition vs. excitation for example). By contrast, it has been difficult to define such internal circuitry in the IC. The papers in this section describe a variety of approaches, anatomical, molecular, and physiological to this question and contribute to our emerging understanding of IC's internal and external organization.

The next 6 chapters describe analyses of the molecular characteristics of IC neurons in relationship to function. These include papers on the role of ion channels in generating responses of IC neurons and on the effects of mutations, aging, and damage to the auditory system. Such insults change the expression of genes and produce a variety of functional consequences for the representation of sound.

The final 13 chapters describe the encoding of sound in the IC, especially in its ascending pathways. These include analyses of responses to sound, convergence of auditory and other inputs in the IC, and analyses of emergent properties like stimulus-specific adaptation. The bat auditory system has long provided fertile ground for auditory research, because of the relatively well-defined computations needed for sonar processing. Almost half of the chapters on sound encoding deal with the bat IC, especially the role of inhibition in determining response selectivity, delay tuning, and duration tuning.

The study of the IC is too large a topic to produce a satisfactory overall view in a collection of papers of the size of this one. However, these papers do represent the main trends in current research on the IC and make clear some of the most important outstanding problems. It is hoped that they will inspire and guide the next steps in working out this critical part of the auditory system.

## Conflict of interest statement

The authors declare that the research was conducted in the absence of any commercial or financial relationships that could be construed as a potential conflict of interest.

